# simplePHENOTYPES: SIMulation of pleiotropic, linked and epistatic phenotypes

**DOI:** 10.1186/s12859-020-03804-y

**Published:** 2020-10-31

**Authors:** Samuel B. Fernandes, Alexander E. Lipka

**Affiliations:** Department of Crop Sciences, University of Illinois at Urbana-Champaign, Urbana, 61801 USA

**Keywords:** Quantitative genetics, Multivariate data, Correlated traits, Computer simulation

## Abstract

**Background:**

Advances in genotyping and phenotyping techniques have enabled the acquisition of a great amount of data. Consequently, there is an interest in multivariate statistical analyses that identify genomic regions likely to contain causal mutations affecting multiple traits (i.e., pleiotropy). As the demand for multivariate analyses increases, it is imperative that optimal tools are available to assess their performance. To facilitate the testing and validation of these multivariate approaches, we developed simplePHENOTYPES, an R/CRAN package that simulates pleiotropy, partial pleiotropy, and spurious pleiotropy in a wide range of genetic architectures, including additive, dominance and epistatic models.

**Results:**

We illustrate simplePHENOTYPES’ ability to simulate thousands of phenotypes in less than one minute. We then provide two vignettes illustrating how to simulate sets of correlated traits in simplePHENOTYPES. Finally, we demonstrate the use of results from simplePHENOTYPES in a standard GWAS software, as well as the equivalence of simulated phenotypes from simplePHENOTYPES and other packages with similar capabilities.

**Conclusions:**

simplePHENOTYPES is a R/CRAN package that makes it possible to simulate multiple traits controlled by loci with varying degrees of pleiotropy. Its ability to interface with both commonly-used marker data formats and downstream quantitative genetics software and packages should facilitate a rigorous assessment of both existing and emerging statistical GWAS and GS approaches. simplePHENOTYPES is also available at https://github.com/samuelbfernandes/simplePHENOTYPES.

## Background

The wealth of data available from high-throughput phenotyping platforms used in modern agronomic experiments enables unprecedented study into the genomic underpinnings of genotype-to-phenotype relationships [1, 2]. In particular, these data make it possible to gain insight into the simultaneous contributions of genomic loci to multiple traits, a phenomenon known as pleiotropy [3]. To ensure that accurate inferences are being made from these data, the most appropriate statistical approaches must be used. One avenue towards assessing the performance of such approaches is to simulate correlated traits in which the pleiotropic and non-pleiotropic quantitative trait nucleotides (QTNs) underlying the genomic sources of phenotypic variability are known.

Several useful software packages have been developed to simulate correlated traits [4–6]. However, there is not a single package that enables the user to control for all possible parameters involved in the genetic architecture of complex traits. For instance, to the best of our knowledge, none of the currently available simulation packages allows the user to simulate spurious pleiotropy as defined by [3]. In this work, we present the R/CRAN package simplePHENOTYPES. This package uses real marker data to simulate additive, dominance, and additive x additive epistatic QTN controlling multiple traits via pleiotropy, partial pleiotropy, and a novel implementation of spurious pleiotropy. We maximize simplePHENOTYPES’ utility to the research community by ensuring its compatibility with popular data formats and state-of-the-art GWAS and GS software. Developmental versions and vignettes (i.e., example code for using the demo genotypic data set to simulate specific genetic architectures in simplePHENOTYPES), may be found at https://github.com/samuelbfernandes/simplePHENOTYPES.

## Implementation

**Fig. 1:**
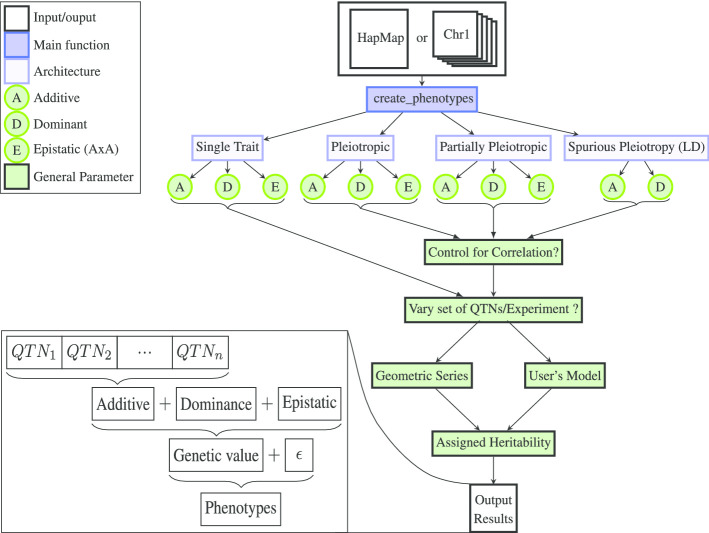
Workflow and main options implemented in simplePHENOTYPES to simulate single and multiple traits

simplePHENOTYPES is capable of simulating traits controlled by a wide range of genetic settings, as depicted in Fig. 1. After reading in biallelic marker data in one of many formats (i.e., Numeric, HapMap, VCF, GDS, Plink Ped/Bed files) into R, the user specifies the desired genetic architecture using the *create_phenotypes()* function. In particular, the user has the option to indicate the degree of pleiotropy among the QTNs, the number of additive and non-additive QTNs, the degree of correlation between multiple traits, and the heritability of each simulated trait.

**Fig. 2:**
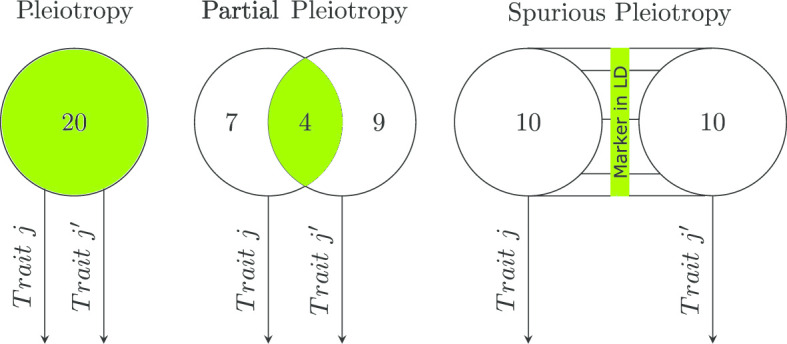
Three scenarios of multi-trait genetic architectures implemented in simplePHENOTYPES to simulate two traits *Trait* *j* and $Trait~j'$ controlled by a pool of 20 QTNs. Pleiotropy: 20 QTNs control both traits; partial pleiotropy: 7, 4, and 9 QTNs controlling *Trait* *j*, both traits, and $Trait~j'$ only, respectively; spurious pleiotropy: 10 independent QTNs controlling each trait but in linkage disequilibrium (LD) with one marker that is also in LD with QTNs from the other trait

When simulating multiple traits, the user has control over whether all QTNs control all traits (pleiotropy), a subset of the QTNs control all traits (partial pleiotropy), or separate QTNs in linkage disequilibrium (LD) control individual traits (spurious pleiotropy). To illustrate these three different scenarios, consider a set of 20 QTNs and two traits (*Trait* *j* and $Trait~j'$), as depicted in Fig. 2. In the pleiotropy scenario, the same 20 QTNs control both of the simulated traits. In the partial pleiotropy scenario, four QTNs control both traits, seven QTNs only control *Trait* *j*, and nine QTNs only control $Trait~j'$. The final scenario, spurious pleiotropy, simulates two correlated traits where ten different QTNs control each trait. Here, simplePHENOTYPES randomly selects ten markers; each of these markers is within a user-specified amount of LD with one QTN controlling *Trait* *j*, as well as another QTN controlling $Trait~j'$. The spurious pleiotropy depicted in Fig. 2 is an implementation of what is defined in Figure 1-f in [3], but simplePHENOTYPES also offers the option to simulate traits where the user-specified maximum LD refers to the maximum LD between each neighboring QTN pairs for the two traits. The user may select between the two LD options by using the *type_of_ld* input parameters of *create_phenotypes()* (see Listing 1 below).

There is substantial flexibility for defining the number of additive and non-additive (i.e., dominance and additive x additive epistasis) QTNs controlling each trait, as well as their effect sizes. These effects may either be manually inputted in a list with a vector of effect sizes for each trait, or as a single value from which simplePHENOTYPES will create a geometric series of effect sizes [7]. The effect sizes will be assigned to each QTN, as described in Table 1. For a given number of QTNs and effect sizes, the user has the option to specify whether or not the same markers are to be the QTNs across all experiments (i.e., replicates of traits with the same genetic architectures) through the *vary_QTN* input parameter of *create_phenotypes()*. The latter option of varying the markers randomly assigned to be QTN should allow the user to have a set of experiments where the QTN allele frequencies and LD in the regions surrounding the QTNs differ for each experiment.

**Table 1:** Effect sizes assigned to each genotype under different genetic models

		genotype		
Model		$-1$	0	1
Additive		$-a^*$	0	*a*
Dominance		0	$d^{\ddagger }$	0
Epistatic	$$\left. \begin{array}{r} -1\\ 0\\ 1 \end{array} \right.$$	$$\left. \begin{array}{r} e^{\dagger }\\ 0\\ -e \end{array} \right.$$	$$\left. \begin{array}{r} 0\\ 0\\ 0 \end{array} \right.$$	$$\left. \begin{array}{r} -e\\ 0\\ e \end{array} \right.$$

${}^{*}$Additive effect; ${}^{\ddagger }$Dominance effect; ${}^{\dagger }$Epistatic (additive x additive) effect

It is possible to both indirectly and directly control the genetic correlation between traits simulated in simplePHENOTYPES. The genetic correlation between traits can be indirectly controlled by assigning different effect sizes to their shared QTNs. Thus, if the same two additive QTNs control two traits, denoted *Trait* *k* and $Trait~k'$, with effect sizes 0.10 and 0.01 for *Trait* *k* and 0.40 and 0.16 for trait $Trait~k'$, these two traits will have a genetic correlation $0< cor(Trait~k, Trait~k')<1$.

Alternatively, simplePHENOTYPES allows users to assign specific Pearson correlations between genetic values of the traits using a process known as whitening/coloring transformation [8]. To illustrate this process, let $\mathbf{Y }$ denote the scaled and centered genetic values (i.e., the cumulative values of the QTNs multiplied by their effect sizes) of each simulated trait. Let $\Sigma$ denote the variance-covariance matrix of the simulated genetic values; since $\mathbf{Y }$ is scaled and centered, $\Sigma$ is equal to a correlation matrix.

To modify $\mathbf{Y }$ so that the desired pairwise correlation between each genetic value is equal to those specified by the user in $\Sigma '$ (which is specified using the *cor* input parameter inside *create_phenotypes()*), the Cholesky decomposition is first applied to $\Sigma$. This phase, called the whitening transformation, calculates $\mathbf{X } = L^{-1/2}\mathbf{Y }$, where *L* is a lower diagonal matrix defined from the Cholesky decomposition of $\Sigma$, i.e., $\Sigma = LL^T$. Each of the genetic values in the resulting vector $\mathbf{X }$ are uncorrelated. To obtain a vector of genetic values $\mathbf{Y}'$ with the pairwise correlations specified in $\Sigma '$, a coloring transformation is applied to $\mathbf{X }$, specifically $\mathbf{Y}' = L'^{1/2}\mathbf{X }$. Similar to the whitening phase, $L'$ is a lower diagonal matrix calculated from $\Sigma ' = L'L'^T$, i.e. the Cholesky decomposition of $\Sigma '$. Finally, the process of centering and scaling the genetic values $\mathbf{Y}'$ is reversed so that these are back on the original scale.

Another feature of simplePHENOTYPES is the ability to specify the heritabilities of each simulated trait. These heritabilities are subsequently used to simulate normally distributed random error terms that represent non-genetic sources of trait variability. By default, these random error terms are independent, but the user may optionally specify a user-defined residual correlation (*cor_res*). Thus, the variance of each simulated trait is equal to the sum of i.) the variance of the genetic values from the QTNs and ii.) the variance of these random error terms. Although the current implementation of simplePHENOTYPES explicitly dichotomizes trait variability into these two sources, we anticipate that future versions will allow the user to specify the contributions of background genetic effects and non-genetic covariates to the simulated traits.

We took several measures to ensure the quality, reproducibility, and accessibility of the results produced by simplePHENOTYPES. Upon completing simulating traits, simplePHENOTYPES will create a log file that will compare the estimated sample heritabilities and (when appropriate) genetic and residual correlations with those specified by the user, as well as confirm details on the specified genetic architecture. In addition, the variance explained by each QTN (calculated prior to the whitening/coloring transformation when the desired pairwise genetic correlations between traits are specified in $\Sigma '$), as well as new marker data without the SNPs selected to be QTNs, are optionally exported into the user’s specified working directory. Finally, the seed numbers used to select QTNs and simulate the random non-genetic error terms for each simulated trait are saved as text files. These, along with genomic information on the markers selected to be QTNs, should facilitate the regeneration of the simulated traits whenever the need arises. The resulting simulated traits are saved in user-specified formats ready for downstream evaluation in external quantitative genetics software packages. Output compatibility includes GEMMA [9] and TASSEL [10]. Similarly, the option of saving the output as an R object ensures quick access by packages such as GAPIT [11] and rrBLUP [12].

## Listings


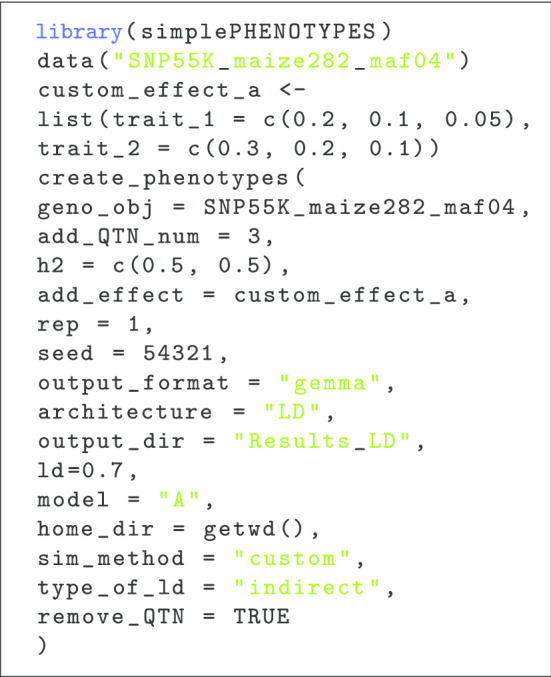

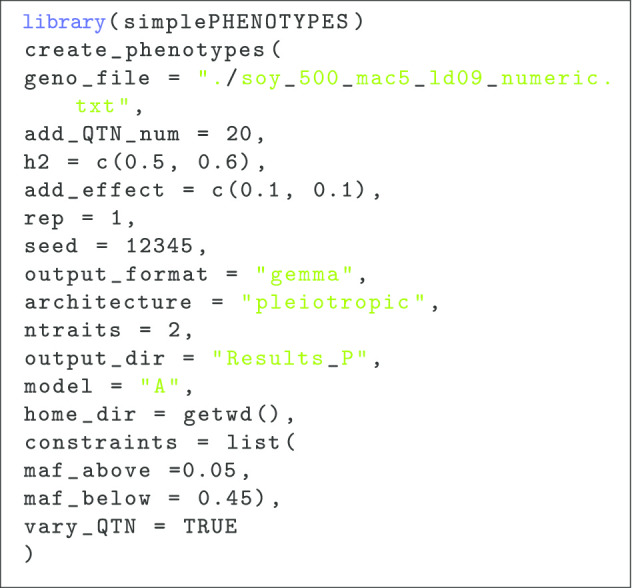


## Results

**Fig. 3:**
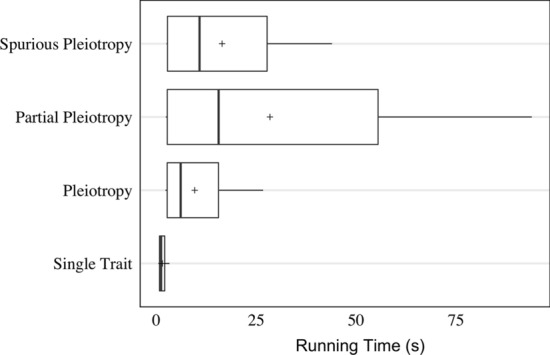
Boxplot of running time, in seconds, required by simplePHENOTYPES to simulate 1, 000 experiments (i.e., replicate traits) under the single trait, pleiotropy, partial pleiotropy, and spurious pleiotropy scenarios. In the latter three scenarios, two traits were simulated for each experiment. This simulation was conducted on a single core of a 2.4 GHz MacBook with 64 GB of RAM. The data set used contained 10, 650 SNPs in numerical format genotyped on 280 maize individuals. The symbol $``+''$ denotes the mean running time of each simulated architecture

simplePHENOTYPES is capable of simulating thousands of experiments on a typical laptop computer in less than one minute (Fig. 3). When we simulated phenotypes from a data set of 10, 650 markers genotyped on 280 maize lines from the Goodman-Buckler Diversity Panel [13, 14] on a single core of a 2.40 GHz MacBook with 64 GB of RAM, the median time to simulate 1, 000 experiments for each evaluated scenario was 2.79 seconds (Fig. 3). In particular, the median completion time for multivariate phenotypes was respectively 6.06, 15.57, and 10.79 seconds for the pleiotropy, partial pleiotropy, and spurious pleiotropy scenarios. Marker data sets in formats that require numericalization (e.g., HapMap formats) will require extra time for the numericalization step. When this same data set was used in a HapMap format, the median completion time was 6.7 seconds (data not shown).

To illustrate the facility of simulating multiple traits in simplePHENOTYPES, we present a sample R script for simulating two traits from the spurious pleiotropy scenario (Listing 1). Here, the same data set of 280 maize lines form the Goodman-Buckler diversity panel is used to simulate two traits controlled by three additive QTNs ($add\_QTN\_num = 3$). Both traits have a heritability of 0.5. The additive effect sizes for the three QTNs of trait 1 (trait 2) are 0.2 (0.3), 0.1 (0.2), and 0.05 (0.1). The LD between a given QTN of each trait and the corresponding common marker ($type\_of\_ld = ``indirect''$) is at maximum $r = 0.7$ ($ld=0.7$).

In a different scenario, we also simulated a pair of traits controlled by 20 pleiotropic QTNs (Listing 2), with a different heritability for each trait and QTNs with a minor allele frequency between 0.05 ($maf\_above =0.05$) and 0.45 ($maf\_below = 0.45$). In this case, we used a soybean data set consisted of a random sample of 500 accessions in maturity groups III and IV downloaded from SoyBase [15] (http://soybase.org/snps/download.php). This data set was comprised of 42, 291 SNPs obtained with the SoySNP50K [16]. We filtered out SNPs with more than missing data conducted an LD pruning step using Plink’s options r2 = 0.9 (–indep-pairwise 100 10 0.9) [17]. We only included SNPs from chromosomal DNA with a minor allele count greater that 5 in this simulation. The final data set was composed of 18, 364 SNPs.

**Fig. 4:**
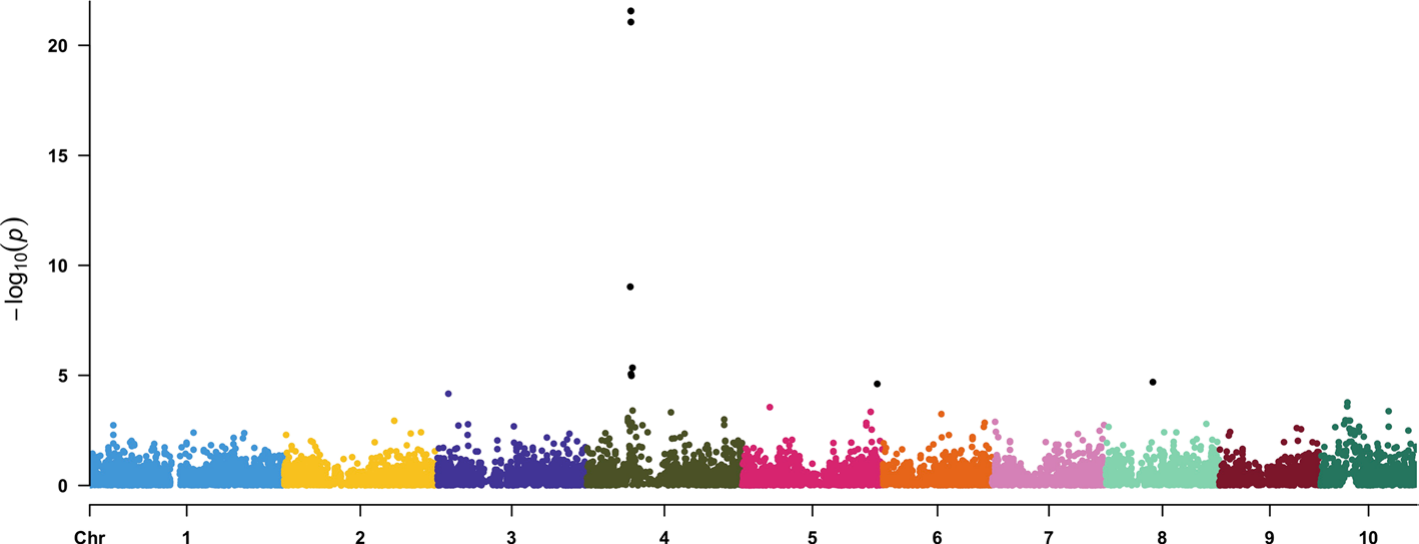
Manhattan plot of a multi-trait GWAS conducted on two phenotypes simulated under the spurious pleiotropy genetic architecture. The X-axis depicts the physical position on the maize genome, while the Y-axis denotes the -log(*P*-value) at each of 10, 650 genome-wide SNPs that were considered in the multi-trait GWAS

**Fig. 5:**
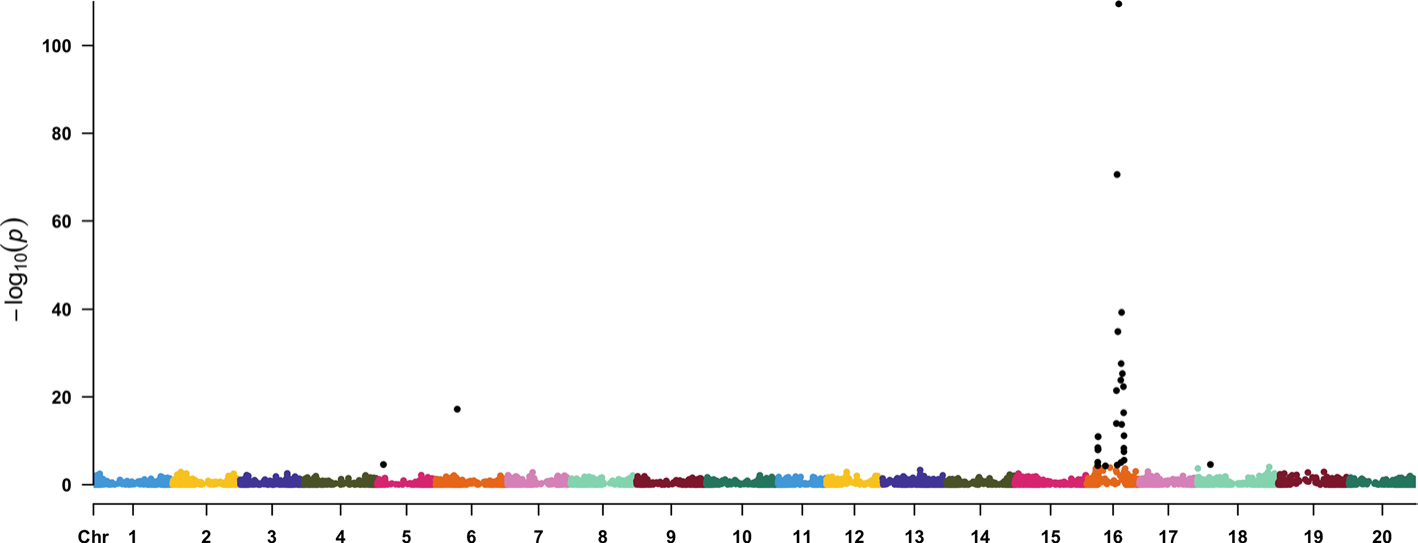
Manhattan plot of a multi-trait GWAS conducted on two phenotypes simulated under the pleiotropy genetic architecture. The X-axis depicts the physical position on the soybean genome, while the Y-axis denotes the -log(*P*-value) at each of 18, 364 genome-wide SNPs that were considered in the multi-trait GWAS

Detailed information on the SNPs selected to be QTNs and a summary of their LD for Listing 1 are presented at Tables 2 and 3, respectively. The details of the SNPs selected for Listing 2 are available on Additional file 1: Table S5. To illustrate the use of these simulated traits in downstream quantitative genetics analysis, we removed the SNPs selected to be QTNs in Listing 1 from the marker data set ($remove\_QTN = TRUE$) and conducted a multi-trait GWAS in an external software package, specifically GEMMA [18] (Fig. 4). The statistical model used in this package is a multivariate version of the unified mixed linear model [19], which includes fixed and random effect covariates to account for spurious associations attributable to population structure and familial relatedness. This same analysis was conducted on traits simulated in Listing 2 (Fig. 5). Other GWAS examples for phenotypes simulated under the pleiotropy and partial pleiotropy scenarios are presented in the section 2 of the Supplementary File. As expected, in all scenarios the peak-associated markers from GWAS were located in the vicinity of the largest-effect QTNs.

**Table 2:** Map and minor allele frequency information of each SNP randomly selected in the sprious pleiotropy simulation presented in Listing 1, as well as those selected to be additive quantitative trait nucleotides (QTNs)

Replication	Marker type	Marker	Allele	Chromosome	Position	$$\hbox {cM}^*$$	$$\hbox {MAF}^{**}$$
1	cause_of_LD	ss196453961	A/G	4	68,182,921	NA	0.50
1	cause_of_LD	ss196498075	A/G	10	87,305,008	NA	0.50
1	cause_of_LD	ss196495963	G/A	10	52,568,307	NA	0.50
1	QTN_upstream	ss196453979	G/A	4	68,369,212	NA	0.50
1	QTN_upstream	ss196498083	A/C	10	87,799,475	NA	0.50
1	QTN_upstream	ss196496034	G/A	10	54,432,827	NA	0.50
1	QTN_downstream	ss196453929	C/A	4	67,678,951	NA	0.50
1	QTN_downstream	ss196498065	A/G	10	87,189,608	NA	0.50
1	QTN_downstream	ss196496813	C/A	10	49,693,682	NA	0.50

A value of $''1''$ under the column labeled “Replication” indicates that these were the markers selected for the first experiment (i.e., the first replicate trait) for this genetic architecture

$^*$ centimorgan (This example does not contain information on genetic linkage).

$$^{**}$$ Marker data filtered by minor allele frequency (MAF) $$> 0.40$$

**Table 3:** Summary linkage disequilibrium information on quantitative trait nucleotide of different traits

Replication	Marker causing $$\hbox {LD}^*$$	input LD (absolute value)	Actual LD with $$\hbox {QTN}^{**}$$ of Trait 1	Actual LD with QTN of Trait 2	QTN for Trait 1	QTN for Trait 2	LD between QTNs
1	ss196453961	0.70	0.16	0.11	ss196453929	ss196453979	0.11
1	ss196498075	0.70	0.24	0.45	ss196498065	ss196498083	$-0.35$
1	ss196495963	0.70	0.66	0.62	ss196496813	ss196496034	0.62

$$^*$$Linkage Disequilibrium; $$^{**}$$Quantitative Trait Nucleotide

To demonstrate the equivalence of simulated trait results between simplePHENOTYPES and similar packages, we compared traits simulated by simplePHENOTYPES to those from AlphaSimR [6], SimPhe [20], and PhenotypeSimulator [4] when equivalent genetic architectures were specified. Due to differences between simplePHENOTYPES and PhenotypeSimulator concerning specific input parameters and implementations used for trait simulation, we compared both packages’ ability to simulate traits controlled by two pleiotropic QTNs with additive effects. Additionally, we compared simplePHENOTYPES to AlphaSimR and SimPhe for traits controlled by either i.) two pleiotropic QTNs with dominance effects, and ii.) one pleiotropic QTN with additive x additive epistatic effects. We showed that when traits with the same QTNs and effect sizes where simulated under a heritability of 1, the simulated traits from simplePHENOTYPES were identical to the other software packages. These identical results suggest that simplePHENOYPTES calculates the genetic contributions of simulated traits in a similar manner to AlphaSimR, SimPhe, and PhenotypeSimulator. Please see the Supplementary File for the code used for this benchmarking.

## Conclusions

simplePHENOTYPES makes it possible to simulate multiple traits controlled by loci with varying degrees of pleiotropy. Its ability to interface with both commonly-used marker data formats and downstream quantitative genetics software and packages should facilitate the rigorous assessment of both existing and emerging statistical GWAS and GS approaches.

## Availability and requirements

- Project Name: simplePHENOTYPES
- Project Home page: https://github.com/samuelbfernandes/simplePHENOTYPES
- Operating system: Tested on Linux, Windows and macOS
- Programming languages: R
- Other requirements: R 3.5 or higher
- License: MIT license
- Any restrictions to use by non-academics: No (free software)

## Supplementary information

**Additional file 1.** Description of the R code used for benchmarking analysis, GWAS conducted on the simulated data sets, and to estimate the run time of simplePHENOTYPES.

## Data Availability

The R code for running simplePHENOTYPES is available at https://github.com/samuelbfernandes/ simplePHENOTYPES, as well as on R/CRAN https://cran.r-project.org/web/packages/simplePHENOTYPES/index.html. The specific R scripts to run the benchmarking study, examples of using simplePHENOTYPES to run a GWAS, and the code used to estimate running time are presented in the supplementary material at: https://github.com/samuelbfernandes/simplePHENOTYPES/blob/master/vignettes/Supplementary.pdf. The soybean SNP data set used in Listing 2 is available at: http://soybase.org/snps/download.php.

## Abbreviations

LD: Linkage disequilibrium
QTN: Quantitative trait nucleotide

## References

[1] Yang W, Guo Z, Huang C, Duan L, Chen G, Jiang N, Fang W, Feng H, Xie W, Lian X, Wang G, Luo Q, Zhang Q, Liu Q, Xiong L (2014). Combining high-throughput phenotyping and genome-wide association studies to reveal natural genetic variation in rice. Nat Commun.

[2] Singh D, Wang X, Kumar U, Gao L, Noor M, Imtiaz M, Singh RP, Poland J (2019). High-throughput phenotyping enabled genetic dissection of crop lodging in wheat. Front Plant Sci.

[3] Solovieff N, Cotsapas C, Lee PH, Purcell SM, Smoller JW (2013). Pleiotropy in complex traits: challenges and strategies. Nat Rev Genet.

[4] Meyer HV, Birney E (2018). Phenotype simulator: a comprehensive framework for simulating multi-trait, multi-locus genotype to phenotype relationships. Bioinformatics.

[5] Porter HF, O’Reilly PF (2017). Multivariate simulation framework reveals performance of multi-trait GWAS methods. Sci Rep.

[6] Faux A-M, Gorjanc G, Gaynor RC, Battagin M, Edwards SM, Wilson DL, Hearne SJ, Gonen S, Hickey JM (2016). Alphasim: software for breeding program simulation. The Plant Genome.

[7] Fisher RA (1930). The genetical theory of natural selection.

[8] Novak L, Vorechovsky M (2018). Generalization of coloring linear transformation. Trans VSB Techn Univ Ostrava Civil Eng Ser.

[9] Zhou X, Stephens M (2012). Genome-wide efficient mixed-model analysis for association studies. Nat Genet.

[10] Bradbury PJ, Zhang Z, Kroon DE, Casstevens TM, Ramdoss Y, Buckler ES (2007). TASSEL: software for association mapping of complex traits in diverse samples. Bioinformatics.

[11] Lipka AE, Tian F, Wang Q, Peiffer J, Li M, Bradbury PJ, Gore MA, Buckler ES, Zhang Z (2012). GAPIT: genome association and prediction integrated tool. Bioinformatics.

[12] Endelman JB (2011). Ridge regression and other kernels for genomic selection with R package rrBLUP. Plant Genome J.

[13] Cook JP, McMullen MD, Holland JB, Tian F, Bradbury P, Ross-Ibarra J, Buckler ES, Flint-Garcia SA (2012). Genetic architecture of maize kernel composition in the nested association mapping and inbred association panels. Plant Physiol.

[14] Flint-Garcia SA, Thuillet A-C, Yu J, Pressoir G, Romero SM, Mitchell SE, Doebley J, Kresovich S, Goodman MM, Buckler ES. Maize association population: a high-resolution platform for quantitative trait locus dissection. The Plant Journal. 2005;44(6):1054–64. 10.1111/j.1365-313X.2005.02591.x. https://onlinelibrary.wiley.com/doi/pdf/10.1111/j.1365-313X.2005.02591.x 10.1111/j.1365-313X.2005.02591.x 16359397

[15] Song Q, Hyten DL, Jia G, Quigley CV, Fickus EW, Nelson RL, Cregan PB (2015). Fingerprinting soybean germplasm and its utility in genomic research. G3: Genes|Genom|Genet.

[16] Song Q, Hyten DL, Jia G, Quigley CV, Fickus EW, Nelson RL, Cregan PB (2013). Development and evaluation of SoySNP50K, a high-density genotyping array for soybean. PLoS ONE.

[17] Purcell S, Neale B, Todd-Brown K, Thomas L, Ferreira MAR, Bender D, Maller J, Sklar P, de Bakker PIW, Daly MJ, Sham PC (2007). PLINK: a tool set for whole-genome association and population-based linkage analyses. Am J Hum Genet.

[18] Zhou X, Stephens M (2014). Efficient multivariate linear mixed model algorithms for genome-wide association studies. Nat Genet.

[19] Yu J, Pressoir G, Briggs WH, Bi IV, Yamasaki M, Doebley JF, McMullen MD, Gaut BS, Nielsen DM, Holland JB (2006). A unified mixed-model method for association mapping that accounts for multiple levels of relatedness. Nat Genet.

[20] Jiang B, Pütz B. Tools to simulate phenotype(s) with epistatic interaction 2018. https://cran.r-project.org/web/packages/SimPhe/SimPhe.pdf.

